# Pseudoperipheral palsy: a case of subcortical infarction imitating peripheral neuropathy

**DOI:** 10.1186/s12883-015-0409-y

**Published:** 2015-08-25

**Authors:** Mirza Jusufovic, Astrid Lygren, Anne Hege Aamodt, Bård Nedregaard, Emilia Kerty

**Affiliations:** Dept of Neurology, Oslo University Hospital, Oslo, Norway; Dept of Radiology, Oslo University Hospital, Oslo, Norway; Institute of Clinical Medicine, University of Oslo, Oslo, Norway; Dept of Psychiatry, Akershus University Hospital, Lørenskog, Norway

**Keywords:** Hand knob area, Peripheral motor nerve deficits, Stroke, Cerebral angiitis

## Abstract

**Background:**

Vascular damage in the central hand knob area can mimic peripheral motor nerve deficits.

**Case presentation:**

We describe the case of a woman presenting with apparent peripheral neuropathy. Brain magnetic resonance imaging and computed tomography angiography revealed an infarct in the precentral hand knob area, with significant stenosis in the right proximal middle cerebral artery trunk. Subsequent 3-Tesla magnetic resonance imaging of the brain suggested cerebral angiitis. The patient experienced improved hand function following combined glucocorticoid and cyclophosphamide treatment.

**Conclusion:**

Vascular damage in the hand knob area should be considered when evaluating peripheral motor nerve deficits in the presence of normal nerve conduction velocities. The diagnosis of cerebral angiitis remains a major challenge for clinicians.

## Background

Vascular damage involving the central hand control network [1] can produce focal weakness of the fingers, with ulnar presentation [2], and/or a radial/medial distribution [3]. Vascular pathology in the hand knob area should be considered when evaluating peripheral motor nerve deficits in the presence of normal nerve conduction velocities. Here we report a case of subcortical infarct in the hand knob area, suggestive of cerebral angiitis, that presented as apparent peripheral neuropathy.

## Case presentation

A 44-year-old, right-handed female presented with sudden hand motor deficits in her left hand. There was no trauma to the arm, she used no medications and she had no vascular risk factors.

**Fig. 1:**
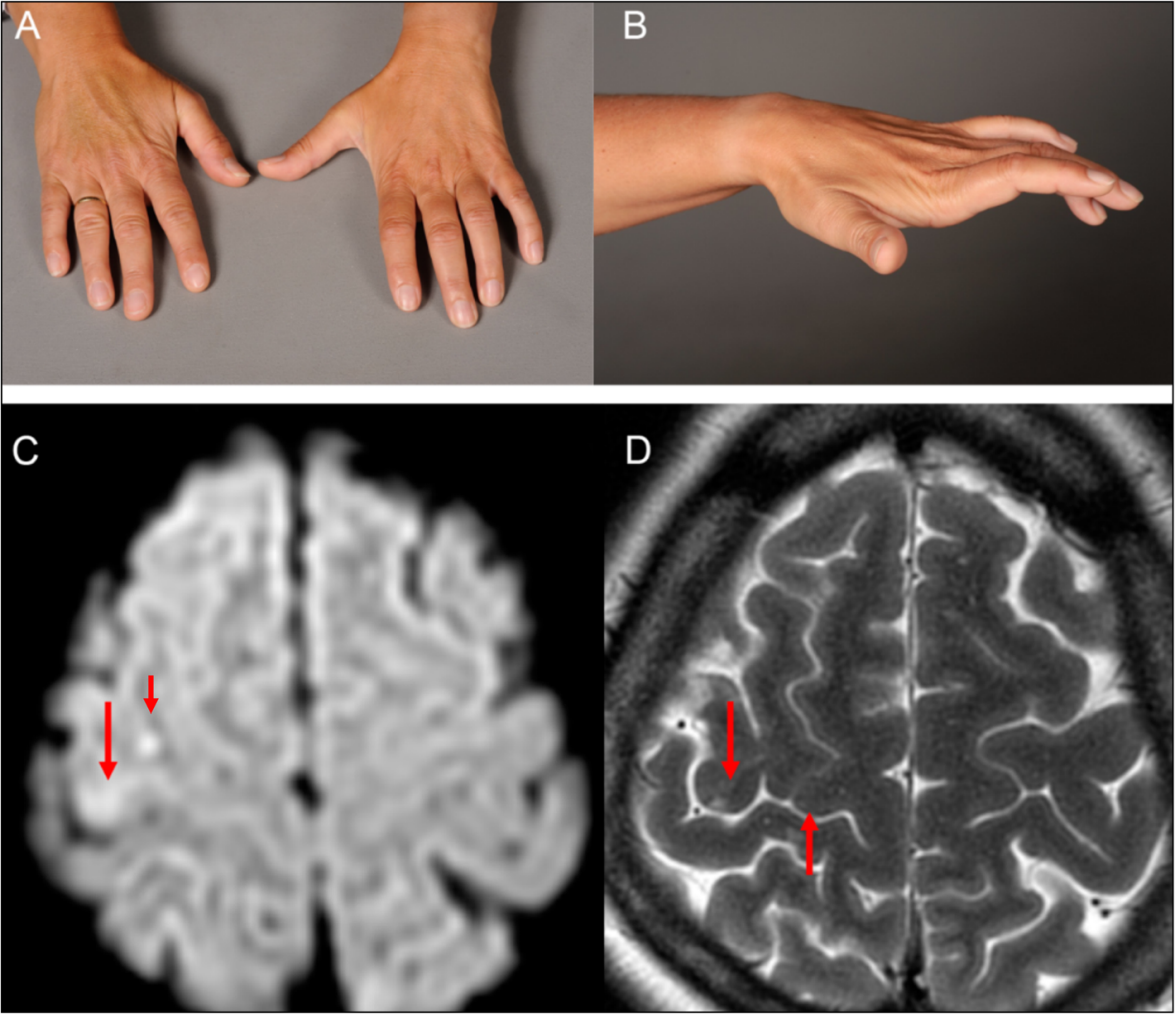
Clinical photographs of the left hand and MRI of the brain. **a**, **b**. Clinical photographs showing claw hand deformity due to infarct in the hand knob area. **c**. Axial diffusion- and, **d**, T2-weighted MRI showing hyperintense signals (upper arrows on **c**) in the right precentral gyrus near the central sulcus (lower arrow on **d**), indicating an infarct in the hand knob area (upper arrow on **d**)

Examination revealed severe motor deficits of the left hand with extension of the three ulnar fingers and wrist, muscle atrophy in the first dorsal interosseous muscle, and claw hand deformity without sensory deficits (Fig. 1a, b). According to the Medical Research Council scale examination revealed grade 1 in the left wrist flexor, grade 4 in the left wrist extensor, grade 3 in the left fingers flexor and grade 4 in the left fingers extensor. Adduction and abduction of the left fingers were also severely impaired (grade 3). Deep tendon reflexes were mildly brisker ipsilateral to the affected hand. No Babinski sign was observed.

Cervical computed tomography (CT) was unremarkable. Peripheral motor nerve conduction velocities were normal. Small, discrete ischemic lesions appeared hyperintense on diffusion-weighted magnetic resonance imaging (MRI) sequence (upper arrows on Fig. 1c). The infarct was located in the right posterior part of the precentral hand knob area (upper arrow on 1d), near the central sulcus (lower arrow on Fig. 1d). CT angiography showed significant stenosis in the right proximal middle cerebral artery (MCA) trunk.

Transesophageal echocardiogram and carotid ultrasound did not suggest an embolic source. Hypercoagulable screening and levels of markers specific to systemic vasculitis were all normal. An embolism arising from the ipsilateral MCA stenosis was subsequently considered as the etiology of the patient’s symptoms, and she received acetylsalicylic acid and clopidogrel for secondary stroke prevention.

**Fig. 2:**
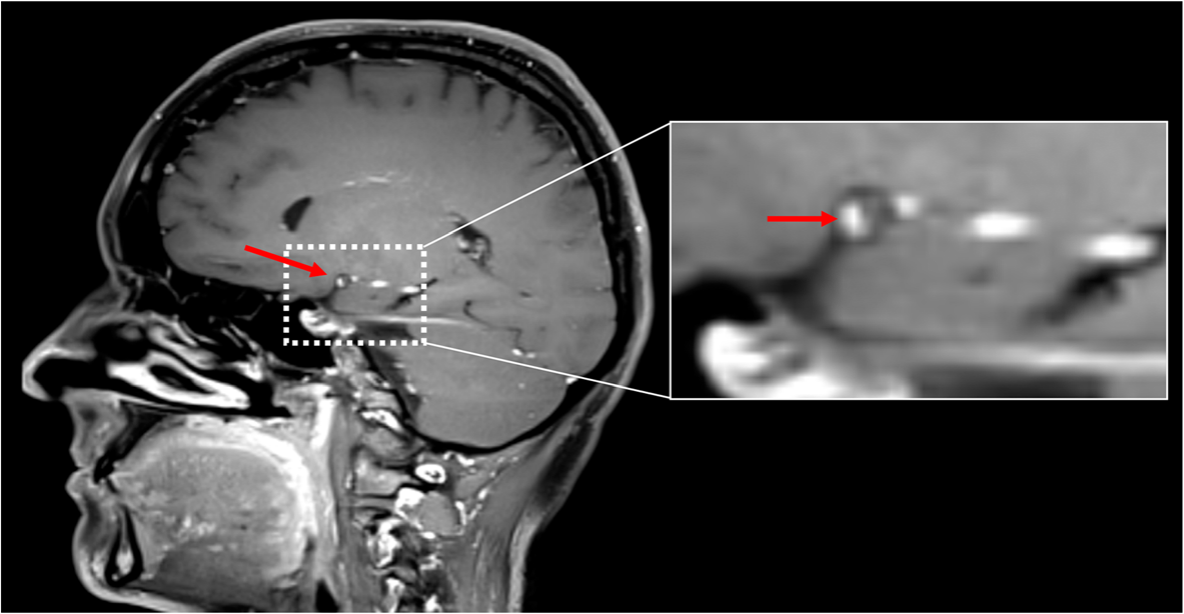
3-Tesla MRI of the brain. T1-weighted (volumetric T1 turbo spin echo) 3-Tesla MRI of the brain with fat suppression and gadolinium contrast showing focal vessel wall enhancement (arrow) of the right proximal middle cerebral artery

**Fig. 3:**
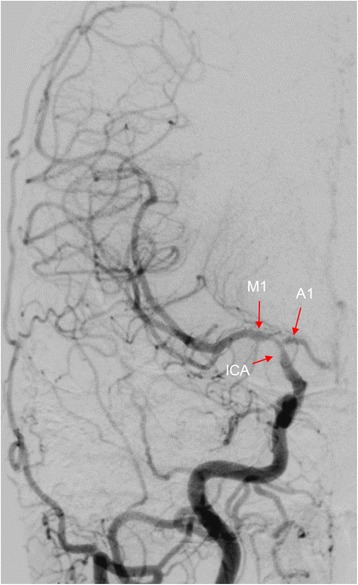
Digital subtraction angiography of the brain. Multiple stenoses at the internal carotid artery (ICA), middle cerebral artery, M1 segment, and anterior cerebral artery, A1 segment were seen on digital subtraction angiography

At the 2-month follow-up, the patient complained of fatigue and progressive headaches. Cerebrospinal fluid analysis was normal. Follow-up 3-Tesla MRI with gadolinium contrast revealed focal wall enhancement in the right proximal MCA (Fig. 2), indicating cerebral angiitis. Multiple stenoses at the same location were seen on digital subtraction angiography (DSA) (Fig. 3). Brain biopsy was not performed.

Cyclophosphamide infusions were administered with glucocorticoids over the subsequent 15 weeks. Left-hand motor function improved, aside from her left fifth finger.

## Discussion

It is known that pure motor nerve deficits resulting from acute stroke can occur in the central hand control network [2], but also angular gyrus [4], the ventroposterolateral nuclei of thalamus [5], internal capsule, corona radiate, pontine base and/or ventromedial medulla. It is hypothesised that discrete functional cortical areas for each finger exist and are sequentially arranged [6], which means that finger movements are controlled by a highly distributed network rather than by functionally and spatially discrete groups of neurons controlling each finger [7]. This is also supported by the findings of one study, in which none of the patients showed hand motor deficits limited strictly to one or a few fingers, but rather both radial and ulnar sided hand motor deficits in varying degrees. The authors suggested this to be a characteristic form of finger motor deficits due to a cortical lesion which is important to differentiate the clinical picture from lesions at other locations [8]. Clinicoradiological signs observed in our patient exhibiting pseudoperipheral neuropathy due to small subcortical infarction well support motor hand area in the motor homunculus in the hand area.

With a muscle hand atrophy, one should also look for compression of the ventral roots or of the anterior horns of grey matter in the cervical regions. The muscle atrophy in such cases is due to a longstanding lower motor neuron lesion and is often accompanied by weakness and fasciculation of the muscles innervated by the affected segments. No radiological evidence for cervical compression was found in our patient.

Functional MRI techniques suggest that the hand knob area, which controls hand motor function [9], is located in the posterior part of the precentral gyrus, near the central sulcus [9–11].

CT of the brain is often insufficient to diagnose the majority of ischemic lesions in the hand knob area, mostly due to the small lesional size and the subcortical location. Diffusion-weighted MRI is more sensitive to confirm the involvement of the precentral knob, and should be included as part of the standard evaluation. The treatment of an infarct in the hand knob area is dependent of the suspected etiology, ranging from arterio-arterial embolism to atherosclerotic infarcts [12]. However, little is known about the underlying pathological mechanisms of this stroke entity [12].

Diagnosis of cerebral angiitis is challenging. Brain biopsy remains the gold standard [13]; however, positive DSA, MRI and cerebrospinal fluid findings are sufficient to diagnose «probable» cerebral angiitis [13, 14]. Recent 3-Tesla MRI studies using increased magnetic field strengths have identified distinct vasculitic patterns, with arterial wall thickening and enhancement in both proximal and distal small intracranial vessels [15–17], that can remain stable for more than 12 months (median follow-up 13.5 months) [16]. 3-Tesla MRI to detect arterial wall inflammation may therefore become the favored criterion for diagnosing cerebral angiitis [17]. Nevertheless, other causes of arterial wall enhancement (e.g., atherosclerosis, radiation vasculopathy, infection, and vasospasm) must be excluded, and studies in a wider range of disorders are required to properly define the role of 3-Tesla MRI in the diagnostic process [17]. It is also possible that our patient had pseudoperipheral palsy due to cerebral angiitis, which was not detected by routine nerve conduction studies.

Although long-term data are scarce, the early-phase prognosis of similar cases is typically good [12], possibly due to the functions carried out by the damaged cortical area being resumed via the recruitment of adjacent areas [18].

## Conclusions

The current case underlines that vascular pathology in the hand knob area can cause claw hand deformity, leading the unwary clinician to suspect peripheral nerve problems. However, a subcortical hand knob infarct can imitate peripheral motor nerve deficits, and is easily overlooked. To best of our knowledge, an infaction in the hand knob area associated with cerebral angiitis has not been previously reported.

## Consent

Written informed consent was obtained from the patient for publication of this Case report and any accompanying images. A copy of the written consent is available for review by the Editor of this journal.

## Abbreviations

CT: Computed tomography
DSA: Digital subtraction angiography
ICA: Internal carotid artery
MCA: Middle cerebral artery
MRI: Magnetic resonance imaging
